# Targeting NK Cells to Enhance Melanoma Response to Immunotherapies

**DOI:** 10.3390/cancers13061363

**Published:** 2021-03-17

**Authors:** Hansol Lee, Inês Pires Da Silva, Umaimainthan Palendira, Richard A. Scolyer, Georgina V. Long, James S. Wilmott

**Affiliations:** 1 Melanoma Institute Australia, The University of Sydney, Sydney 2006, Australia; hansol.lee@sydney.edu.au (H.L.); Ines.Silva@melanoma.org.au (I.P.D.S.); m.palendira@centenary.org.au (U.P.); Richard.Scolyer@health.nsw.gov.au (R.A.S.); james.wilmott@sydney.edu.au (J.S.W.); 2 Faculty of Medicine and Health Sciences, The University of Sydney, Sydney 2006, Australia; 3 Department of Infectious Diseases and Immunology, The Charles Perkins Centre, School of Medical Sciences, The University of Sydney, Sydney 2006, Australia; 4 Department of Tissue Pathology and Diagnostic Oncology, Royal Prince Alfred Hospital and NSW Health Pathology, Sydney 2006, Australia; 5 Department of Medical Oncology, Royal North Shore Hospital and Mater Hospital, Sydney 2065, Australia; 6 Sydney Medical School, The University of Sydney, Sydney 2006, Australia

**Keywords:** melanoma, natural killer cells, innate immune system, immune checkpoint inhibitors

## Abstract

**Simple Summary:**

NK cells are innate immune cells that form one of the initial responses to infections and cancers. There have been increasing number of studies investigating the anti-tumor effects of NK cells. Immunotherapy targeting NK cell may enhance the therapeutic efficacy of current immunotherapy regimes. Through pro-inflammatory cytokine production, enhancing B cell production of antibodies, facilitate and activate dendritic cells, activate T cells and participating in anti-tumor immunity through the granzyme B pathway and antibody-dependent cellular cytotoxicity, the versatility of NK cells provides an attractive immunotherapy option. This review highlights NK cell biology, NK cell antitumor immunity, NK cell immune evasive mechanisms and novel immunotherapies that aim to target NK cells.

**Abstract:**

Natural killer (NK) cells are a key component of an innate immune system. They are important not only in initiating, but also in augmenting adaptive immune responses. NK cell activation is mediated by a carefully orchestrated balance between the signals from inhibitory and activating NK cell receptors. NK cells are potent producers of proinflammatory cytokines and are also able to elicit strong antitumor responses through secretion of perforin and granzyme B. Tumors can develop many mechanisms to evade NK cell antitumor responses, such as upregulating ligands for inhibitory receptors, secreting anti-inflammatory cytokines and recruiting immunosuppressive cells. Enhancing NK cell responses will likely augment the effectiveness of immunotherapies, and strategies to accomplish this are currently being evaluated in clinical trials. A comprehensive understanding of NK cell biology will likely provide additional opportunities to further leverage the antitumor effects of NK cells. In this review, we therefore sought to highlight NK cell biology, tumor evasion of NK cells and clinical trials that target NK cells.

## 1. Introduction

Along with innate lymphoid cell 1 (ILC1), natural killer (NK) cells are a part of the group 1 innate lymphoid cells (ILC) family that differentiate from common lymphoid progenitor cells [1]. Unlike ILC1, which display more of a T helper cell-like phenotype and are noncytotoxic, NK cells are cytotoxic [2]. NK cells not only detect and destroy viruses and other pathogens, but also cells undergoing early malignant transformation, without previous priming, hence they are members of the innate immune system. While NK cells constitute only 5–15% of total lymphocytes in peripheral blood [3], their innate ability to detect and target transformed cells (e.g., tumor cells) makes them key cells in antitumor immune surveillance. Mature circulating NK cells carry granules of granzymes and perforins and store transcript for IFN-γ, which they can utilize to destroy tumor cells at first contact in minutes [4]. The innate ability of NK cells to release a potent cytotoxic response has contributed to increasing interest in their utility as a therapeutic target in cancer.

The contribution of NK cells in the immune response against cancer was recorded as early as the 1970s [5,6]. One of the first NK cell studies investigated the effects of NK cell depletion on the growth of melanoma and found that depleted NK cells in melanoma-bearing mice resulted in higher tumor burden compared to non-NK cell depleted mice [7]. Subsequent studies also found that NK cells produce IFN-γ, which is a critical ingredient for the activation and recruitment of cytotoxic T lymphocytes (CTLs) [8]. These cytokines are also essential for an effective adaptive immune response against cancer through macrophage and dendritic cells (DC) activation, M2-macrophage and CD4+ T cell differentiation and suppression of myeloid derived suppressor cells (MDSC) and Tregs (extensively reviewed in [9,10]). This review focuses on the importance of NK cells in mediating an effective anticancer immune response in the context of melanoma and opportunities to overcome tumor resistance to modern immunotherapies.

### 1.1. NK Cells Subtypes

**Figure 1:**
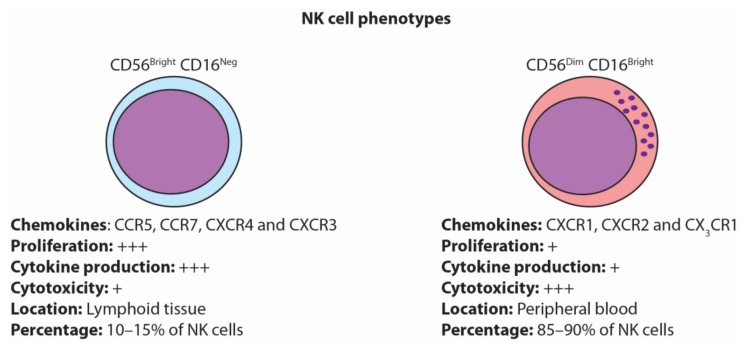
Natural killer (NK) cell phenotypes. NK cells are generally classified as either the immature CD56^bright^ CD16^neg^ or the mature CD56^dim^ CD16^bright^.

NK cells are generally characterized into two major subsets based on their expression of CD56 (adhesion molecule) and CD16 (Fc gamma receptor). NK cells are often classified as CD56^bright^CD16^neg^ or CD56^dim^CD16^bright^, which represent functionally distinct NK cell phenotypes [11] (Figure 1). CD56^bright^CD16^neg^ NK cells represent 10–15% of the total NK cell population and are mainly present in lymph nodes (~75%). They are potent producers of cytokines, especially IFN-γ, and have poor cytolytic capacity [11]. In contrast, CD56^dim^CD16^bright^ NK cells represent 90% of peripheral blood NK cells and possess strong cytolytic capacity but are relatively poor producers of cytokines [12].

### 1.2. NK Cell Activation

NK cell activation is highly dynamic and dependent upon the cumulative effect of activating and inhibitory signals [13]. NK cells have a repertoire of receptors that send activating or inhibitory signals into the NK cell [14]. The ligands for NK cell activating receptors are only upregulated upon cellular stress such as a viral infection or malignant transformation [15]. On the other hand, the ligands for NK cell inhibitory receptors are largely confined to the various heavy chains of the MHC Class I molecule expressed on all healthy nucleated cells [16]. Changes in this balance, for example, the absence of MHC class I expression, are considered abnormal and push NK cells towards an activated state. When the cumulative effect of activating and inhibitory signals surpasses the NK cell activating threshold, NK cell activation occurs [17,18].

Under normal circumstances, healthy nucleated cells, engagement between MHC class I and various killer cell immunoglobulin-like receptors (KIRs) and/or NKG2A/CD94 provide inhibitory signals to NK cells [16]. When this is coupled with the lack of activating receptor stimulation, NK cells are rendered anergic [14]. However, when the target cell becomes virally or malignantly transformed, the cell may downregulate MHC class I and/or upregulate ligands that are stimulated upon cellular stress. The lack of inhibitory receptor activation due to MHC class I downregulation, coupled with large signals being propagated by NK cell-activating receptors, due to the upregulation of stress ligands, cause NK cell activation [14]. Therefore, for NK cells to perform effector functions, stimulation of multiple activating receptors is required to overcome the threshold of activation.

## 2. NK Cell-Activating Receptors and Their Ligands

### 2.1. Natural Cytotoxicity Receptors (NCRs)

NCRs are NK cell-activating receptors that that play an important role in NK cell-mediated tumor killing [19]. There are three NCRs expressed on human NK cells: NCR1 (NKp46/CD335), NCR2 (NKp44/CD336) and NCR3 (NKp30/CD337). While NCR1 and NCR3 are constitutively expressed on resting NK cells [20,21,22], NCR2 is only induced upon NK cell activation [23].

The diversity of NCR ligands enables NK cells to effectively detect microbial infections or tumor cells. NCR ligands can be microbial proteins that are presented on their plasma membrane or proteins that are upregulated upon cellular stress. However, due to difficulties in copurifying NCR with NCR ligands, many tumoral NCR ligands remain unknown [24]. Using NCR-Ig fusion proteins that target the extracellular domain of NKp30, NKp44 and NKp46, the density of various NCR ligands was quantified without knowing the exact identity of all the NCR ligands [25]. In metastatic melanoma, NKp30 ligands were minimally expressed [25], while NKp44 and NKp46 ligands had heterogenous expression that seemed to depend on tumor heterogeneity and site of disease [25,26].

There are several NCR ligands that have been discovered in melanoma cells. B7-H6 is a protein that is not expressed on healthy cells and is a ligand for NKp30; however, upon malignant transformation, the activation of the proto-oncogene *Myc* causes upregulation of B7-H6 [27]. B7-H6 is expressed on the surface of melanoma cells [28], and this protein is shown to be susceptible to membrane shedding by metalloproteases [29]. In fact, B7-H6 is detectable at high levels in the serum of melanoma patients, highlighting that B7-H6 shedding may represent an immune evasive mechanism by melanoma cells to evade potential NK cell mediated killing. Human leukocyte antigen-B-associated transcript 3 (BAT3) is another NKp30 ligand that was shown to upregulate surface expression on melanoma cells upon the activation of the pattern recognition receptor, retinoic acid-inducible gene I (RIG-I), and its upregulation led to potent antitumor activity in melanoma mice models [30]. Galectin-3 is another NKp30 ligand that is expressed on melanoma cells [31]. Despite galectin-3 increasing metastatic potential of tumor cells by increasing its motility via metalloproteinase regulation [32], strong galectin-3 expression in melanoma patients was associated with improved survival [31].

### 2.2. DNAX Accessory Molecule-1 (DNAM-1/CD226)

The expression of the activating receptor DNAM-1 varies on NK cells, with NK cells that highly express DNAM-1 producing higher levels of proinflammatory cytokines and chemokines (e.g., IFN-g, IL-6, GM-CSF and CCL5), having increased proliferation rates, and being responsive to IL-15 stimulation [33]. DNAM-1 has two known ligands, CD155/PVR and CD112/nectin-2 [34,35,36]. CD155 is expressed in the majority of melanomas, while only 26% of melanomas express CD112 [37]. Interestingly, CD155 expression was seen in the majority of primary and metastatic melanoma, with CD155 showing significant correlation with known negative melanoma prognostic markers such as lymph node involvement and Breslow thickness of the primary tumor [38]. Tumor-expressed CD155 can bind to DNAM-1 on NK cells and enhance the ability of the NK cell to effectively suppress and kill tumor cells in a DNAM-1-dependent manner [26,39]. In fact, CD155 expression on melanomas has also shown to be associated with a poorer response of metastatic melanoma patients to anti-PD-1 monotherapy and combination anti-PD-1 and CTLA-4 [40]. CD155 also exists in a soluble form secreted by tumor cells [41], which impacts NK cell functionality by impairing NK cell activation via DNAM-1 [42]. In fact, patients with advanced gastric cancer had significantly higher levels of soluble CD155 (sCD155), and sCD155 levels directly correlated with tumor burden [43]. The secretion of sCD155 is not restricted to gastric cancer, as other cancers such as breast, prostate and melanoma also secrete sCD155 [44]. Unlike other soluble NK cell ligands, sCD155 is not a result of protease activity [44], but rather due to the CD155 isotype that lacks the transmembrane domain [41].

### 2.3. Natural-Killer Group 2 Member D (NKG2D)

NKG2D is an activating receptor that belongs to the family of C-type lectin-like receptors and is expressed on all NK cells, along with a subset of activated dendritic and CD8^+^ T cells [45]. In NK cells, NKG2D expression correlates with degranulation and IFN-g expression in NK cells [46]. NKG2D receptors have eight known ligands that are upregulated upon cellular stress, which include MICA, MICB and ULBP1, 2, 3, 4, 5 and 6. Of all the NKG2D ligands, MICA was shown to be highly expressed in melanoma [47]. However, these ligands can also be shed off the tumor surface through disulphide isomerase (ERp5) and several proteases belonging to disintegrin and metalloproteinase (ADAM) and matrix metalloproteinase (MMP) families [48,49,50,51]. In fact, blocking MICA and MICB shedding from the tumor surface reduced melanoma metastasis and improved the antitumor immunity in an NKG2D- and CD16-dependent manner [52]. The absence of MICB and ULBP-1 in baseline serum, signifying that MICB and ULBP-1 had reduced shedding, is significantly correlated with improved survival in patients with metastatic melanoma treated with immune checkpoint blockade [53].

### 2.4. CD16a

There are two isotypes of CD16: CD16a (F_c_γRIIIa) and CD16b (F_c_γRIIIb). CD16a is expressed by several immune cell types including NK cells, macrophages, monocytes, neutrophils and T cells, while CD16b is exclusively expressed by neutrophils [54]. In NK cells, CD16a binds to the F_c_ portion of the immunoglobulins IgG1 and IgG3, and, to a minimal extent, with IgG2 and IgG4 [55], and mediates antibody-dependent cell-mediated cytotoxicity (ADCC). Unlike other activating NK cell receptors, the engagement of CD16a to IgG is sufficient for NK cells to release perforin and granzyme B, leading to tumor cell death [56,57,58]. In addition to ADCC, CD16-IgG ligation decreases CD16 surface expression, increases IFN-g production and upregulates CD54 on NK cells [59].

CD16a is a low-affinity Fc receptor; however, a recent study has uncovered the diversity of 234 CD16 polymorphisms which alter the affinity of CD16 to IgG between individuals. Certain polymorphisms also improved the reactivity of NK cells to all four IgG isotypes (IgG1, IgG2, IgG3 and IgG4). For instance, the presence of homozygous valine (V/V) or heterozygous valine/phenylalanine (V/F) at amino acid 158 enhances CD16 binding affinity to IgG1 and IgG3 compared to homozygous phenylalanine (F/F) phenotype [60,61]. As a result, a lower antibody concentration is needed to effectively activate CD16 with a V158V or V158F polymorphism [59]. In this study, 62% of participants had either V158F (55%) or V158V (7%) CD16 polymorphisms that increased CD16 binding affinity, while 38% had F158F polymorphism, which caused CD16 binding affinity to be lower than that of V158F and V158V [60]. This highlights that a significant proportion of society has polymorphisms, at least at amino acid 158, that increase CD16 binding affinity. Further studies are required to delineate whether multiple CD16 polymorphisms are present and whether CD16 binding affinities contribute to a patient’s response to immunotherapies.

### 2.5. Other NK Cell Activating Molecules

CD69 is a membrane-bound type II C-type lectin and is associated with NK cell activation [62]. Not only is CD69 a marker of NK cell activation, it also plays a functional role in improving NK cell cytotoxicity and various effector functions [63]. CD69 has various ligands, including galectin-1 [62], S100-A8/S100-A9 [62] and myosin light chains 9 and 10 [64]. CD69 induction is mediated by IL-2, IFN-α, CD16 cross-linking and activation of NK cell receptors [65], which stimulated signaling via PTK [65,66] or PKC pathways [65]. While PKC blockade resulted in the abolishment of CD69 induction by unknown NK cell activating ligands, PKC blockade did not affect CD69 induction by IL-2, IFN-α and CD16 cross-linking, PTK blockade abrogated CD69 expression in NK cells stimulated by IL-2, IFN-α, CD16 cross-linking and activation of NK cell-activating ligands [65]. CD69 was shown in many studies to induce NK cell cytotoxicity against tumor cells [67,68,69,70] and that CD69 blockade reduced NK cell cytotoxic capacity to the same level as not activated NK cells [67]. Interestingly, anti-CD69 agonists not only improved NK cell cytotoxicity, but also decreased TGF-β1 expression, increased TGF-α expression, induced NK cell proliferation, upregulated CD25 and ICAM-1 expression [63]. This highlights the multifaceted role of CD69 in regulating NK cell effector functions and may be an attractive future immunotherapy target to improve NK cell responsiveness.

CD44 is a cell surface glycoprotein that not only indicates NK cell activation, but also induces cytokine production and enhances NK cell-mediated cytotoxicity [71,72,73]. CD44 is constitutively expressed on NK cells and when it binds with hyaluronan [72], the main ligand for CD44, coupled with the recruitment of serine-threonine, tyrosine and PI3K signalling, perforin- and granzyme B-dependent NK cell cytotoxicity occurs [73]. In fact, mAb activating CD44 was shown to improve NK cell cytotoxicity [74]. Not only is CD44 implicated in NK cell cytotoxicity, CD44–CD44 ligand interaction upregulates CD69 expression [71], TNF-α production [71], IFN-γ production [72], enhanced CD16-mediated ADCC [75] and the adhesion molecules LFA-3 and LFA-1 [75]. Interestingly, the upregulation of adhesion molecules enhances the binding of NK cells to target cells [75].

## 3. NK Cell Inhibitory Receptors and Their Ligands

### 3.1. Killer-Cell Immunoglobulin-Like Receptors (KIRs)

Although the majority of KIRs are inhibitory receptors, some are activating receptors; *KIR* gene family encode receptors that recognize HLA-A, B, C or G, a major component of MHC class I. The 14 *KIR* genes and 2 pseudogenes enable great diversity through *KIR* haplotype and allelic variations [76,77], thus resulting in approximately 1000 allelic variations [78]. Inhibitory KIRs have long cytoplasmic tails (L) accompanied by immunoreceptor tyrosine-based inhibitory motifs (ITIMs), while activating KIRs have a short cytoplasmic tail (S) accompanied by immunoreceptor tyrosine-based activating motifs (ITAMs), with the exception of KIR2DL4, which is an activating KIR that has a long cytoplasmic tail [76,79].

The function of activating KIRs is not well known and activating KIRs are thought to have evolved from inhibitory KIRs [80]. Many NK cells do not possess activating KIRs, suggesting that these receptors are not essential for NK cell functionality [81]. NK cells activated by KIR2DL4 function to stimulate NK cell cytokine production rather than cytotoxicity, highlighting that activating KIRs may shape NK cell responses [79]. In contrast, inhibitory KIRs play an essential role not only in mediating inhibitory signals to suppress NK cell activation, but also in educating NK cells by tuning the threshold for NK cell activation. Without inhibitory KIRs, NK cells become unresponsive [82]. MHC class I downregulation is a tumor evasive mechanism [83] that frequently occurs, and through KIRs, NK cells possess the ability to recognize and eradicate these MHC class I-deficient tumor cells.

Despite several studies, there are no consistent results that highlight an association between KIR receptors with response or prognosis. The expression of KIR2DS5 (activating KIR) was shown to be lower in rapidly progressing patients with advanced melanoma compared to those who progressed slowly [84]. Within the same cohort, stage III or IV melanoma patients had higher expression of KIR2DL2 compared to stage I melanoma patients [84]. In a different study, KIR2DL3 was found to be a protective marker for nodular and ulcerated melanoma [85]. More recently, no NK cell KIR genotypes or KIR/KIR-ligand combinations were shown not to correlate with response to anti-PD-1 therapy [86]. The inconsistencies in attributing certain KIRs with response may be due to great haplotype and allelic diversity within and between ethnicities [87] or may potentially reflect the level of NK cell reactivity via NK cell education, as KIR receptors are known to play an important role in NK cell education [78]. Monoclonal antibody blocking of KIR receptors may improve NK cell functionality and improve patient survival, as demonstrated by the promising effects of KIR-HLA mismatch NK cells in the treatment of leukemia [88].

### 3.2. CD94/NKG2A

NKG2A/CD94 is a heterodimeric receptor that induces inhibitory signals; high expression is associated with an NK cell exhaustion phenotype [89]. NKG2A/CD94 recognizes the nonclassical MHC class I molecule, HLA-E [90]. HLA-E is not recognized by KIR receptors, has limited polymorphism and is expressed in low levels in normal tissue [91,92]. Generally, HLA-E is absent or expressed in low amounts by melanomas. However, IFN-γ significantly upregulates the expression of HLA-E on the tumor cell surface [93]. Given that NK cells are known potent producers of IFN-γ [94], it is likely that the presence of NK cells could cause an upregulation of HLA-E on melanoma cells. This in turn enables the tumor to become resistant to NK cell killing. This resistance to NK cell killing can be effectively reversed through NKG2A blockade [95]. NKG2A blockade has been shown to improve NK cell cytotoxicity against tumor cells, but it also increases IFN-γ production [96].

### 3.3. TACTILE (CD96)

TACTILE (CD96) has been shown to bind CD155 with higher affinity than DNAM-1 [97]. However, the role of TACTILE is unclear, as some studies have shown it to be associated with NK cell activation [98], while others demonstrated that the presence of ITIM-like motifs compete with DNAM-1, which consequently regulates NK cell-mediated cytotoxicity [97,99].

### 3.4. Other Immune Checkpoint Receptors

Programmed cell death protein 1 (PD-1), cytotoxic T-lymphocyte-associated protein 4 (CTLA-4), T-cell immunoglobulin and mucin domain containing-3 (TIM-3) and T cell immunoreceptor with Ig and ITIM domains (TIGIT) are all immune checkpoint receptors that are known to be expressed by T cells, and when stimulated, inhibit T cell activation. Like T cells, PD-1, CTLA-4, TIM-3 and TIGIT are also expressed on NK cells, whereby the expression of these receptors suppresses NK cell response. This will be explored later in this review.

## 4. NK Cell-Mediated Antitumor Immunity

### 4.1. NK Cell Recruitment to the Tumor Microenvironment

Chemokine receptor profiles contribute to the function of NK cells and govern their location within the body [100]. CD56^bright^ and CD56^dim^ express different chemokine receptor profiles that contribute to their phenotype [100]. CD56^bright^ NK cells constitutively express the receptors CCR7, CCR5, CXCR4 and CXCR3 [100] that attract CD56^bright^ NK cells to secondary lymphoid organs [101]. Conversely, CD56^dim^ NK cells uniquely express CX_3_CR1, CXCR2 and CXCR1 [100,102,103]. However, CD56^dim^ NK cells can express lower levels of CXCR3, CXCR4 and CCR7 in the presence of IL-18 [104]. CCR7 can also be acquired on CD56^dim^ NK cells via trogocytosis with mature DCs in a KIR2DS1-dependent and IL-18-independent manner [105,106]. In malignancies, several chemokines, including CCL5, CCL27 and CX3CL1 [107], can be produced by tumor cells, which in turn contributes to the recruitment of NK cells. In B16-F10 mouse model of melanoma, genetic silencing of CCL5 in tumor cells completely abrogated NK cell recruitment to the tumor microenvironment (TME) [108]. In particular, CX3CL1, a chemokine that recruits CXCR3-expressing CD56^dim^ NK cells, is a positive prognostic factor for patient survival and NK cell infiltration in five different malignancies [109,110,111,112,113]. This suggests that CD56^dim^ NK cells and their chemoattractants are important for tumor control in patients with malignant disease.

### 4.2. Antitumor Effects of NK Cells in the TME

**Figure 2:**
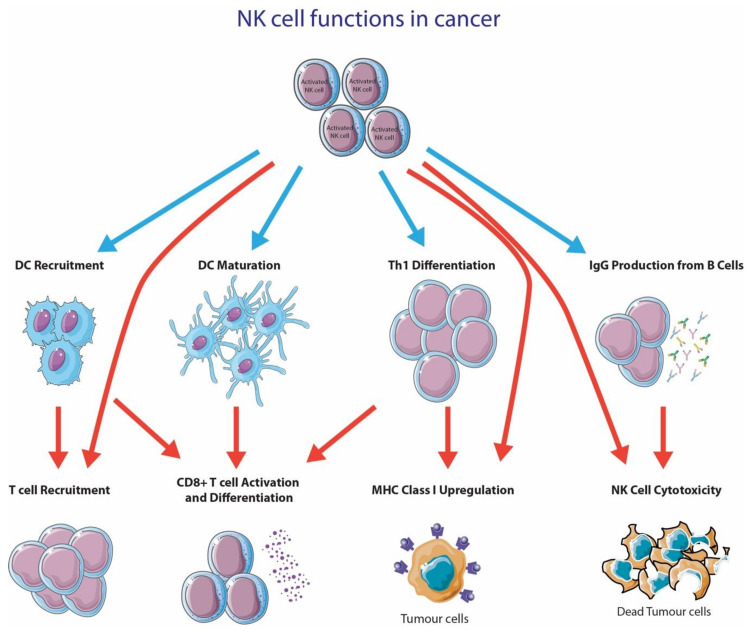
NK cell functions in cancer. This figure was created using Servier Medical Art templates, which are licensed under a Creative Commons Attribution 3.0 Unported License; https://smart.servier.com (accessed 10 October 2020).

NK cells within the TME have many effects on tumor cells (Figure 2); in particular, they have the capacity to directly lyse tumor cells. NK cell-mediated cytotoxicity is initiated by the accumulation of activating signals that overpowers the inhibitory signals. When NK cell cytotoxicity is initiated, NK cells secrete perforin, which forms membrane pores on the target membrane, which would then allow granzyme B to enter the target cell and cause apoptosis through caspase activation [114]. NK cells can also induce apoptosis in its target cell through the interaction between Fas ligand (FasL/CD95L) or TNF-related apoptosis-inducing ligand (TRAIL) with death receptors on the target cell [115].

NK cells also play an important role in augmenting the antitumor capacity of other immune cells. In particular, NK cells produce various proinflammatory cytokines like IFN-γ, GM-CSGF, TNF and many others [116]. NK cells are also implicated to produce T cell recruiting chemokines (IL-8, MIP-1a and RANTES) upon ADCC [117]. One critical cytokine produced by NK cells is IFN-γ, as it has profound effects on a broad range of immune cells [118]. IFN-γ has been shown to enhance tumor antigen presentation on MHC class I [119], reduce angiogenesis [120], induce production of IgG from B cells [121,122], activate macrophages and NK cells [123,124], promote Th1 differentiation [125] and have antiproliferative [126] and apoptotic effects [127].

Recent studies have suggested that NK cells play a critical role in DC recruitment to the tumor microenvironment [107]. DCs are essential for CD8+ T cell activation, which is one of the first steps towards alerting the adaptive immune system for proliferation and the execution of T cell effector functions [128]. The recruitment of Batf3+ DCs (cDC1) was shown to be crucial for CD8+ T cell recruitment [128] and the generation of an immunologically inflamed tumor [129]. Böttcher and colleagues have recently discovered that NK cells played an essential role in recruiting CD8+ T cells into the TME via CCL5 and XCL1 secretion [130]. In fact, several studies have found that tumor-infiltrating NK cells are an important source of XCL1 [130,131]. In fact, the XCL1-XCR1 axis is shown to be exclusively connected to cross-presenting DCs [132,133,134]. The recruitment of XCR1+ cross-presenting DCs via XCL1-secreting NK cells is essential for generating a robust T cell response. In addition to DC recruitment via NK cell-dependent XCL1, NK cells also convert infiltrating monocytes into DCs [135]. This conversion is dependent on direct cell-to-cell contact between NK cells and monocytes, and NK cell-secreted cytokines, GM-CSF and CD154 [135].

### 4.3. NK Cells Are Important for DC Maturation and Activation

DCs are essential innate immune cells that play an important role in bridging the innate and adaptive immune systems. T cells only activate and proliferate when professional antigen presenting cells, like DCs, present antigens on either MHC class I or II to CD4+ or CD8+ T cells, respectively. In the past decade, NK cells have gained significant attention due to their critical role in DC maturation or activation.

Before DC maturation/activation, DCs remain in an immature state, where they are specialized for antigen capture [136,137,138,139]. Immature DCs in cancer are deleterious to effective antitumor immunity [140,141], due to several reasons. Immature DCs lack expression of costimulatory molecules [142] and therefore, when they present antigens, it results in apoptosis [143,144] or anergy [144,145] or development into Treg [146]. However, activated NK cells can lyse immature DCs via NKp30, despite the increased expression of MHC class I [147]. By contrast, mature DCs are protected from NK cell lysis [147], likely due to their expression of NKp30 ligands. Additionally, a hallmark of DC maturation is the upregulation of MHC class I [148]; therefore, the lack of MHC class I expression on immature DCs may also contribute to NK cells selectively lysing immature DCs. Therefore, NK cell-mediated DC editing promotes antigen-specific T cell proliferation, but most importantly, generates a greater protective response during cancer vaccinations [149].

## 5. NK Cell Evasion Mechanisms Utilized by Melanoma

**Figure 3:**
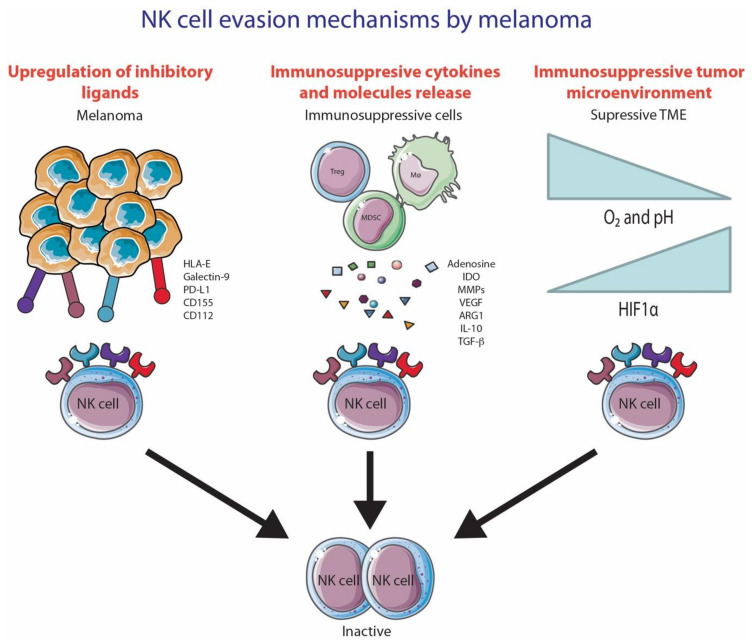
NK cell evasion mechanisms by melanoma. (1) NK cell inhibition due to the upregulation of inhibitory ligands; (2) Secretion of immunosuppressive cytokines/molecules by immunosuppressive cells; (3) Hypoxic tumor microenvironment. This figure was created using Servier Medical Art templates, which are licensed under a Creative Commons Attribution 3.0 Unported License; https://smart.servier.com (accessed 10 October 2020).

Many cancers upregulate the ligands for NK cell-activating receptors during malignant transformation. However, many cancers, including melanoma, develop various ways to evade NK cell recognition and lysis. NK cell evasion can be mediated through cell-to-cell contact with melanoma cells, secretion of cytokines/molecules by tumor cells or by immunosuppressive cells and the creation of a hypoxic tumor microenvironment (Figure 3).

### 5.1. Cell-to-Cell Contact

NK cells have many inhibitory receptors on their cell surface that constantly engage with their environment. Tumor cells are able to upregulate ligands for these inhibitory receptors to inhibit NK cell responses. Regulatory T cells (Tregs) are inhibitory T cells that are recruited to the tumor environment. Tregs inhibit NK cell responses through membrane-bound TGF-b interaction [150] and CTLA-4 [151]. Antigen presenting cells can also anergize NK cells through CD80 or CD86 interaction with CTLA-4 on NK cells [152]. Cell-to-cell contact between tumor-associated fibroblasts can cause the downregulation of an important NK cell-activating receptor, DNAM-1 [153]. However, the mechanism by which this cell-to-cell contact inhibition occurs is unknown.

Ironically, cell-to-cell contact between NK cells and tumor cells has been shown to not only impact the expression of activating receptors, but also NK cell survival via several mechanisms [154,155,156,157]. In one study, NK cell interaction with tumor cells led to NK cell depletion in a caspase- and Fas-independent manner [157]. This depletion was mediated through CD18 and ICAM [157]. In another study, NK cells incubated in IL-2, followed by coculture with leukemia cells, caused NK cell apoptosis [155]. Interestingly, NK cell apoptosis was mediated through CD16 engagement [155] due to the upregulation of Fas on NK cells [156]. The source of FasL causing NK cell apoptosis may be endogenous [154]. In fact, the engagement of NCRs on NK cells with NCR ligand+ tumor cells caused upregulation and secretion of FasL in NK cells. The autologous FasL interacted with Fas and induced NK cell apoptosis through caspase 3 [154]. The CD16 expression level was also shown to decrease after CD16 and CD16 ligand interaction [155,158,159]. Studies have elucidated that matrix metalloprotein (MMP) 25 [159] and ADAM17 as well were responsible for cleaving CD16 from the NK cell membrane. Although ADAM17 [158] is uniformly expressed on NK cells, MMP25 was upregulated on NK cells by IL-2 [159]. Interestingly, upon CD16 engagement, MMP25 was shown to migrate and accumulate at the immunological synapse between the NK and target cell [159].

The down regulation of MHC class I by melanoma has been shown to be a major mechanism of resistance of melanoma patients to anti-PD-1 based immunotherapies [83]. Ironically, tumor cells also upregulate the expression of MHC class I to evade NK cell activation against the tumor. Balsamo and colleagues found that when NK cells are cultured in low effector/target ratios with melanoma cells, the tumor cells upregulate MHC class I, thereby evading the NK cell response [160]. However, this increase in MHC class I expression may improve T cell-mediated killing of melanoma. In that case, tumors have many well-described immunosuppressive mechanisms, many of which are current targets of immune checkpoint inhibitors, such as PD-1 and PD-L1. Therefore, it is likely that tumors alter their immune evasive phenotype to adapt to their immunological challenges.

Lastly, even though it is well established that NK cells are able to target cells that have low MHC expression and/or overexpress the natural ligands of NK cell-activating receptors, chronic stimulation of activating receptors (by abnormally high levels of ligands or abnormally low levels of MHC) have been found to induce NK cell exhaustion [161].

### 5.2. Secretion of Cytokines/Molecules from Tumor Cells and Immunosuppressive Immune Cells

Melanoma cells can respond to an IFN-γ mediated NK cell attack through the secretion of potent immunosuppressive enzymes indoleamine 2,3-dioxgenase (IDO) and prostaglandin E2 (PGE_2_). These enzymes decrease the expression of activating receptors NKp30 and NKG2D on NK cells [162]. IDO is also able to inhibit NK cell functionality, as it catalyzes the production of L-kynurenine, which is known to interfere with IL-2-induced upregulation of NK cell-activating receptors NKp46 and NKG2D [163]. Other immunosuppressive cytokines/molecules secreted by melanoma and known to inhibit NK cell function include adenosine [164], TGF-β [165,166] and IL-10 [167,168].

Through chemokines, tumors are able to recruit immunosuppressive immune cells to the tumor microenvironment, which inhibits NK cell activity. The presence of M2 macrophages, myeloid-derived suppressor cell (MDSC), T regulatory cells (Tregs) and fibroblasts plays a role in suppressing NK cell functions through the secretion of various different molecules and cytokines (extensively reviewed in [169]). Notably, TGF-β is one of many immunosuppressive cytokines/molecules secreted by these suppressive cells, and it has been reported to have an effect in two ways. Firstly, TGF-β can directly decrease the expression of activating receptors NKp30 and NKG2D [34]. Secondly, TGF-β is able to convert NK cells, whether it is CD56^dim^ or CD56^bright^, into a phenotype resembling decidual NK cells (dNK cells), which are poorly cytotoxic and secrete VEGF [170].

In addition, tumor-secreted enzymes are able to shed the activating ligands B7-H6, MICA, MICB and ULBP2 from NK cells through the activity of metalloproteases [29,50,171,172,173]. These ligands have been shown to induce constant stimulation and subsequent endocytosis and degradation of its activating receptors, thereby decreasing NK cell function [48]. In fact, studies have shown that the shedding of activating ligands, such as MICA and UBLP2, is associated with lower survival in stage IV melanoma patients [171,174].

### 5.3. Hypoxic Tumor Microenvironment

The hypoxic conditions created by tumor cells cause alterations both in tumor cells and immune cells, as indicated by the upregulation of HIF-1α (extensively reviewed in [169]). In NK cells, hypoxia causes the activation of autophagy, consequently causing the degradation of granzyme B [175]. Additionally, hypoxia reduces the response of NK cells to proinflammatory cytokines, IL-2, IL-12, IL-15 and IL-21, and consequently inhibits their ability to upregulate activating receptors such as NCRs and NKG2D [176]. Hypoxia affects not only the expression of activating receptors, but also the expression of activating ligands on tumor cells. For instance, hypoxia was shown to downregulate surface expression of MICA, a ligand for the NK cell-activating receptor NKG2D, in a HIF-1a-dependent manner [177].

## 6. NK Cells in the Context of Immunotherapies

There is mounting evidence that NK cells infiltrate various tumors. For instance, NK cells were shown to infiltrate renal cell carcinoma [178,179,180,181,182], melanoma [183,184], breast cancer [185,186,187,188], hepatocellular carcinoma [189,190,191], lung cancer [192,193], prostate cancer [194], bladder cancer [195] and rectal cancer [196]. Although many studies show the presence of intratumoral CD56+ NK cells, studies have also found that intratumoral NK cell infiltration is very low or nonexistent in glioblastoma [197], colorectal cancer (CRC) [198], melanoma [199,200], renal cell carcinoma [157], hepatocellular carcinoma [200] and breast cancer [200]. The reasons behind the discrepancy between tumors with high and low NK cell infiltration are diverse: cohort selection, site of biopsy/sample, the appropriate use of immune markers to identify NK cells and the use of whole slides vs. tissue microarrays (TMAs). In particular, the use of TMA vs. whole slides may significantly influence the scoring of CD56+ NK cells, especially considering that NK cells are not an abundant cell type in the TME [201,202]. In fact, it was shown that TMAs were not an appropriate tool for analyzing less abundant biomarkers and particularly immune cells, and whole slide sections should be used instead [201,202]. Nevertheless, intratumoral NK cell infiltration is shown to be associated with improved overall survival in solid cancers [203,204].

**Figure 4:**
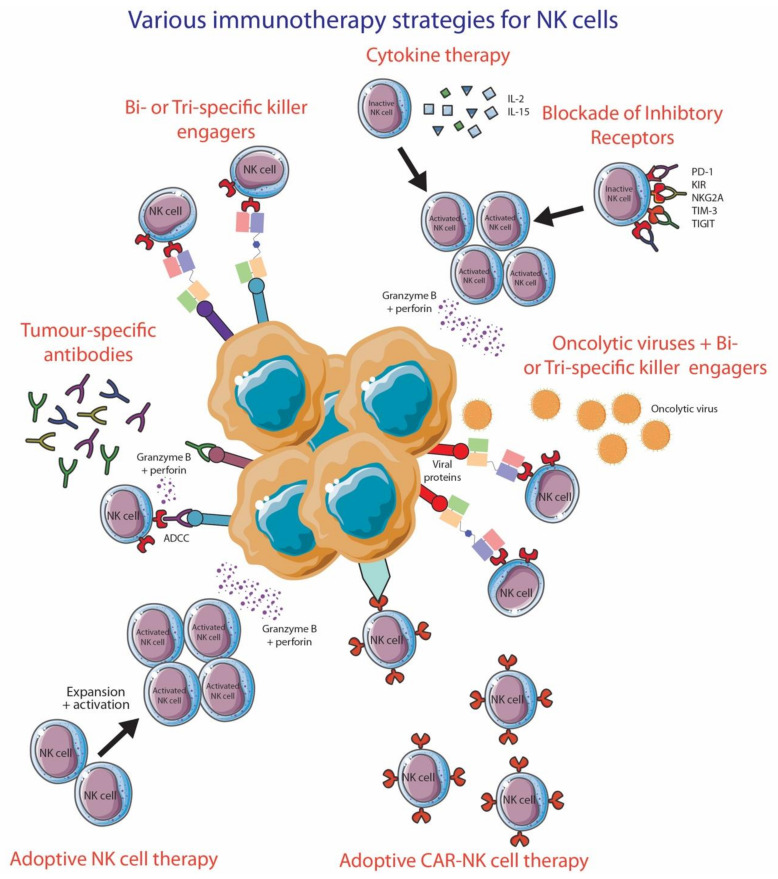
Various immunotherapy strategies to improve NK cell functionality. This figure was created using Servier Medical Art templates, which are licensed under a Creative Commons Attribution 3.0 Unported License; https://smart.servier.com (accessed 10 October 2020).

The diverse functions and importance of NK cells to antitumor therapy have put NK cells as an attractive target for immunotherapy, mainly via blocking inhibitory receptors expressed by these innate immune cells. In this section, we will review the role of NK cells for different treatment strategies in melanoma (Figure 4). There are numerous clinical trials that are attempting to understand the significance of augmenting NK cell responses in the treatment of various malignancies (Table 1).

### 6.1. Anti-PD-1 and Anti-CTLA-4

Anti-PD-1 and anti-CTLA-4 have revolutionized the treatment of metastatic melanoma, and checkpoint inhibitors have become a standard of care for those with advanced disease. Anti-PD-1 and anti-CTLA-4 play a pivotal role in regulating T cell function, but also have a role in NK cell functionality.

CTLA-4 was shown to be expressed on activated mouse NK cells, and CLTA-4 expression inhibits IFN-γ production by NK cells [152]. In fact, the therapeutic benefit of anti-CTLA-4 was partially dependent on NK cells, as the depletion of both NK and CD8+ T cells significantly decreased the therapeutic response to anti-CTLA-4 [205]. There is little data on the expression and impact of CTLA-4 on human NK cells. One study highlighted that patients treated with anti-CTLA-4 induced NK cell activation and enhanced cytotoxicity [206].

PD-1 is another immune checkpoint that is expressed on NK cells upon activation [94]. In vitro studies show that blocking the PD-1 pathway allows T cell exhaustion to be partly overcome, resulting in enhanced proliferation of CD8^+^ and CD4^+^ T cells and increasing their cytokine production [207,208]. NK cells do not express PD-1 as abundantly as T cells, but rather in the range from 1 to 29% [184,209,210], as a proportion of NK cells, with one study showing that 30–40% of NK cells had strong PD-1 upregulation [211], highlighting PD-1 expression heterogeneity on NK cells. However, unlike T cells, PD-1 expression does not induce or imply NK cell exhaustion [211,212]. Given that PD-1 expression is induced on NK cells by IL-2 [213], IL-15 [214] and IL-18 [215], PD-1 expression may represent NK cell activation or maturation status, as demonstrated by a study highlighting that only the mature CD56^dim^ NK cells, and not CD56^bright^, express PD-1 [216]. NK cells have also shown the ability to acquire PD-1 expression via trogocytosis with leukemia cells, which did not depend on PD-1-PD-L1 ligation, highlighting the dynamic ability of NK cells to regulate its immune response [217].

Nevertheless, PD-1 expression on NK cells is implicated in NK cell dysfunction by repressing PI3K/AKT signalling activation in NK cells [218]. PD-1 ligation with PD-L1 led to decreased ability of NK cells to secrete IFN-γ, express granzyme B + perforin and CD107a, a marker indicating NK cell degranulation [213]. Numerous studies have shown that PD-1 blockade improves NK cell activation and cytotoxicity on PD-1+ NK cells and not PD-1- NK cells [209,218,219,220,221]. PD-1 blockade was also shown to significantly increase not only the expression of NK cell-activating receptors NK2GD, NKp44 and NKp30 [220], but also CXCR4 expression on NK cells and the formation of immune complexes between anti-PD-1-treated NK cells and tumor cells [221].

In mouse models of melanoma, NK or CD8+ T cell depletion caused significant reduction in the survival of melanoma when treated with anti-PD-1 + anti-CTLA-4, supporting the hypothesis that NK and CD8+ T cells cooperate with each other to eradicate the tumor in response to these therapies [222]. In fact, CD16+ NK cells were shown to be significantly more abundant in advanced melanoma patients treated with anti-PD-1 therapy [184]. In the same study, Lee and colleagues found that patients with low MHC class I but high intratumoral NK cell density showed superior survival when compared to patients with low MHC class I and low NK cells. This suggests that NK cells possess the ability improve the therapeutic efficacy of anti-PD-1 therapy by potentially eradicating tumors with MHC class I downregulation. In contrast, CD69+ NK cells were shown to negatively correlate in melanoma patients with progression-free survival (PFS) less than 6 months, while CD69+ NK cells were positively correlated with PFS in melanoma patients with PFS greater than 6 months [223]. The discrepancy may be due to other resistance mechanisms that the tumor may employ to evade NK cell recognition and eradication directly [224] or indirectly through the action of other tumor-mediated immune evasion [225]. Subrahmanyam and colleagues found that MIP1β+ CD69+ NK cells were significantly higher in melanoma patients responding to anti-PD-1 therapy [226]. NK cells represent a promising target for immunotherapy to improve the therapeutic efficacy of anti-PD-1 therapy.

### 6.2. Anti-KIR

Many studies have consistently reported that KIRs are upregulated in patients with melanoma, compared to healthy control. In one study, KIR3DL1 and KIR2DL3 were expressed at higher levels on the NK cells of melanoma patients [227], while other studies found an increase in KIR2DL1 [228,229] and KIR2DL2/2DL3 [46,230]. Given that KIRs inhibit NK cell activation, there are several clinical trials underway examining drugs that inhibit KIR, including Lirilumab (ClinicalTrials.gov Identifier: NCT01750580, NCT0174739) and ASP98374 (ClinicalTrials.gov Identifier: NCT03260322).

### 6.3. Anti-NKG2A

NKG2A/CD94 is an inhibitory receptor that is associated with NK cell exhaustion. High NKG2A/CD94 expression on NK cells was associated with poor prognosis of patients with liver cancer [89]. The induction of NKG2A expression may have been stimulated via IL-10 [89]. Nevertheless, in preclinical models, the blockade of NKG2A was shown to improve the antitumor response by NK cells [95,96]. Currently, monalizumab (anti-NKG2A) is in phase 1/2 trials for the treatment of advanced solid tumors (ClinicalTrials.gov Identifier: NCT02671435).

### 6.4. Anti-TIM-3

TIM-3 is another inhibitory receptor that binds to galectin-9 [231], phosphatidylserine on apoptotic cells [232], high mobility group box 1 [233] and carcinoembryonic antigen-related cell adhesion molecule 1 [234]. TIM-3 is associated with an exhausted NK cell phenotype that has a dysfunctional cytotoxic capacity [227,235]. Blocking TIM-3 improves the cytotoxic ability of NK cells [227,236]. High TIM-3 expression was shown to be correlated with poor prognosis in lung adenocarcinoma and metastatic melanoma [227,236]. Currently, there are several phase 1 trials using anti-TIM-3 for advanced solid tumors (ClinicalTrials.gov Identifier: NCT03708328, NCT03489343). Interestingly, in mouse models, the therapeutic effect of anti-PD-1 + anti-TIM-3 was dependent on NK cells bearing MHC class I-deficient tumors [237].

### 6.5. Anti-TIGIT

TIGIT is an immunomodulatory receptor expressed by T cells and NK cells. TIGIT shares the same ligands with DNAM-1 and TACTILE; CD112 (Nectin-2) and CD155 (PVR) [99,238]. Not only does this receptor play a role in inhibiting NK cell responses, it was shown to play a role in NK cell education. In the absence of TIGIT, NK cells are rendered unresponsive to stimuli [238]. There has been increasing interest in anti-TIGIT in clinical trials (ClinicalTrials.gov Identifier: NCT02913313, NCT02676869), and several studies have identified that TIGIT negatively regulates NK cell function by suppressing IFN-γ production and cytotoxicity [97,99,239,240]. Blocking TIGIT was shown to improve NK cell cytotoxicity and slow tumor growth in melanoma mouse models [241]. Several studies have associated TIGIT expression with NK cell exhaustion [241,242]. However, given that TIGIT competes for ligands of the activating receptor, DNAM-1, it is likely that high expression of TIGIT sequesters the ligands from DNAM-1.

### 6.6. Adoptive NK Cell Transfer

Chimeric antigen receptor (CAR)-modified T cells have become an effective immunotherapy option. CAR-T cell therapy has shown remarkable outcomes in B cell lymphoma, with complete response rates between 88 and 93% (sample size ranging from 16 to 30) [243,244,245]. However, severe toxicity (i.e., cytokine release syndrome), graft-versus-host disease, autoimmunity and various T cell resistance mechanisms (i.e., MHC class I loss) are still a concerning issue [246]. CAR-NK cell therapy is an emerging field that has shown great patient safety, limited/no toxicity and is not affected by resistance mechanisms seen in CAR-T cell therapy [247]. In addition to excellent patient safety, CAR-NK cells are not restricted by antigen specificity or strict antigen presentation rules; in fact, CAR-NK cells have numerous activation pathways that ensure effective NK cell activation to tumor targets [224]. CAR-NK cell therapy is a rapidly evolving field, as evidenced by numerous clinical trials using CAR-NK cell therapy in multiple myeloma (BCMA; ClinicalTrials.gov Identifier: NCT03940833), pancreatic cancer (ROBO1; ClinicalTrials.gov Identifier: NCT03941457), glioblastoma (HER2; ClinicalTrials.gov Identifier: NCT03383978), B-cell acute lymphoblastic leukemia (CD19; ClinicalTrials.gov Identifier: NCT00995137) and various solid tumors (NKG2D ligands; ClinicalTrials.gov Identifier: NCT03940820). For instance, a recent phase 1 and 2 clinical trial (CAR-NK cell therapy) highlighted that patients with CD19-positive lymphoid tumors did not develop cytokine release syndrome, neurotoxicity, graft-versus-host disease or increases in inflammatory cytokines, and the maximum tolerated dose was not reached [248]. Most importantly, 73% had a complete response (*n* = 11) with the infused CAR-NK cells persisting at low levels for a minimum of 12 months [248]. When CAR-NK cells were used in a phase 1 and 2 trial involving lung-, pancreatic-, colon- and ovarian cancer, the treatment was well-tolerated and no adverse reactions were seen [246]; no further results were reported. However, adoptive cell therapy has historically had variable success in solid tumors due to the highly complex and immunosuppressive tumor microenvironment [225,249]. While many unique immunosuppressive mechanisms are largely absent in hematological cancers, the tumor microenvironment in solid tumors has numerous and complicated resistance mechanisms that make it difficult for infused CAR-NK cells to not only reach the target site but, prevent itself from being overwhelmed by immunosuppressive signals [225]. It is likely that effective CAR-NK cell therapy is dependent on multiple immunotherapy treatments that address solid tumor-specific immunosuppressive mechanisms such as PD-1, hypoxia or MHC class I downregulation.

### 6.7. Bispecific Killer Cell Engagers (BiKE) and Trispecific Killer Cell Engagers (TriKE)

NK cells possess the ability to become activated and efficiently remove tumor cells through ADCC. A major component of this is the CD16 receptor, which is expressed on mature and differentiated NK cells. CD16 engagement to Fc portions of antibodies opsonized on target cells is sufficient for NK cell release of perforin and granzyme [56,57,58]. In addition to CD16-mediated killing of tumor cells, CD16 engagement also leads to NK cell activation, proliferation [250] and the production of proinflammatory cytokines such as IFN-γ [158]. This makes CD16 an attractive target for immunotherapy as the treatment with monoclonal antibodies, particularly monoclonal antibodies that target tumor-specific antigens or antigens overly expressed on tumor cells enable those cells to become tagged for clearance by CD16+ NK cells. In fact, NK cell-mediated ADCC in tumor-antigen targeting monoclonal antibodies, such as rituximab, contributes to the response of treated patients [251,252]. However, a limitation of CD16 is that differences in CD16 allotype affinity to antibodies mean variations in antibodies being able to elicit appropriate ADCC in NK cells. BiKE and TriKE are able to bypass this by being able to bind to CD16 with strong affinity to elicit ADCC.

BiKEs and TriKEs are emerging as promising immunotherapy options to fully utilize the ADCC capacity of NK cells to effectively eradicate tumor cells. BiKEs consist of two single-chain variable fragments (scFv); scFv is only the antigen binding region of an antibody [253]. One scFv is specific for CD16, while the other scFv is specific for an antigen, in this instance, tumor-specific antigen, and these two scFv are connected together by a short peptide that enables great flexibility [253]. When BiKEs bind to their targets, ADCC and proinflammatory cytokine production occurs in NK cells. TriKEs are very similar to BiKEs, but have three scFv, where the additional scFv is specific to another antigen of choice or another NK cell receptor, or contains a cytokine, like IL-15, integrated in the peptide linker between two scFv.

IL-15 has been chosen as a superior candidate over IL-2 due to its superior safety and toxicity profile and benefits to NK cell activation, proliferation and effector functions. Despite being an important proinflammatory cytokine, administering IL-2 to patients is highly toxic and potentially life-threatening. The most common cause of IL-2 cytotoxicity is vascular leak syndrome that causes hypovolemia and fluid accumulation in the interstitial tissue [254]. IL-2 may also cause the activation of the immunosuppressive CD25+ Treg that can inhibit NK cell function [255]. In contrast, IL-15 does not cause vascular leak syndrome or activate Tregs. However, in a phase I clinical trial, patients with various solid tumors had eight serious adverse events (bowel ischemia, pneumonitis, papilledema, uveitis and grade 3 hypotension), and further studies are required to evaluate the safety and toxicity of IL-15 in humans [256]. In saying this, within the same study, IL-15 dramatically induced a 358-fold expansion of circulating CD56^bright^NK cells [257]. IL-15 was also shown to be important in regulating NK cell survival [258], improving NK cell cytotoxicity through the upregulation of NKG2D, perforin and TRAIL [259] and enhancing NK cell-mediated ADCC [260]. These enhancing effects of IL-15 on NK cells highlight that IL-15 is an attractive candidate to be used as a linker in TriKE.

TriKE containing IL-15 is shown to be a superior therapy option to BiKE [261]. When compared to BiKE, TriKE elicited superior NK cell cytotoxicity, increased IFN-γ and TNFα production, NK cell survival and proliferation [261] when leukemia cell lines were used as targets for NK cells. Within the same study, in vivo studies found that TriKEs had superior antitumor activity and sustained NK cell survival for at least three weeks. The same effects were seen when ovarian, prostate and lung cancers were used, highlighting that TriKEs are effective against solid tumors [262]. Although TriKEs used in these studies contained IL-15, and IL-15 elicited severe toxicity (bowel ischemia, pneumonitis, papilledema, uveitis and grade 3 hypotension) and death in humans [256], IL-15-incorporting TriKEs showed no observable detrimental effects in mice [263].

A caveat with BiKE and TriKE therapy is that they target overexpressing tumor antigens that are also expressed on healthy cells. Additionally, not all tumor cells may express tumor antigens, with some tumors not expressing those antigens at all. In a large number of cancers, a targetable tumor antigen has not yet been identified, making BiKE and TriKE therapy a challenge. A potential option could be to use oncolytic viruses, since a natural aspect of the viral lifecycle is the expression of viral proteins on the cell surface. Here, BiKE and TriKE therapy could be used to target viral protein expression on tumor cells after the delivery of oncolytic viruses [264]. In melanoma, talimogene laherparepvec (T-VEC) has been used in several clinical trials [265]. T-VEC is a herpes simplex virus-1 (HSV-1) that has been genetically engineered to target melanoma cells. Here, HSV-1 proteins could be used as targets to BiKE and TriKE and, given that HSV-1 proteins are not expressed by human cells, this may enable a safer and more effective immunotherapy target compared to targeting tumor antigens.

## 7. Conclusions

NK cells are multifunctional immune cells that ensure injury and disease are effectively addressed. They play numerous concurrent roles such as efficiently participating in tumor eradication. Recent studies have revealed that NK cells play an essential role in activating both CD4+ and CD8+ T cell responses through intricate NK-DC interactions. In malignancies, NK cells may play a critical role in efficiently coordinating the adaptive immune system. Given the success of CAR-NK cells in hematological malignancies, the use of CAR-NK cells in solid tumors should concurrently consider addressing the diverse resistance mechanisms seen in solid tumors for optimal therapeutic efficacy.

## Figures and Tables

**Table 1:** Table of clinical trials involving drugs that optimize NK cell functionality.

	Trial Name/Reference	Population	Drugs	Phase	Recruitment Status
*Anti-KIR*	BMS-986015 NCT01750580	Advanced solid tumours	(1) Lirilumab + Ipilimumab (*n* = 22)	1	Completed
*-*	NCT01714739	Advanced refractory solid tumours	(1) Nivolumab + Lirilumab (2) Nivolumab ± Lirilumab (3) Nivolumab + Lirilumab (4) Nivolumab + Ipilimumab +Lirilumab (*n* = 337)	1/2	Active, not recruiting
*-*	NCT03260322	Advanced solid tumours	(1) Participants enrolled in escalation or expansion cohorts, receiving IV ASP98374 on day 1 of every 3-week cycle (up to maximum of 8 dose strengths) (*n* = 363) (2) Participants enrolled in escalation or expansion cohorts, receiving IV ASP98374 and pembrolizumab on day 1 of every 3-week cycle (up to maximum of 5 dose strengths and 1 fixed dose strength of pembrolizumab)	1	Recruiting
*Anti-TIGIT*	NCT02913313	Advanced or metastatic solid tumours	(1) Dose escalation of BMS-986207 (*n* = 170) (2) Dose escalation of BMS-986207 + Nivolumab (3) Expansion of BMS-986207 (4) Expansion of BMS-986207 + Nivolumab	1/2	Recruiting
*-*	NCT02676869	Unresectable or metastatic melanoma	(1) IMP321 dose escalation: administered fortnightly in addition to pembrolizumab (*n* = 24)	1	Active, not recruiting
*Anti-TIM3*	NCT03708328	Advanced and/or metastatic solid tumours	(1) RO7121661 administered in treatment cycles once every 2 weeks. Dose escalation will be carried out according to a modified continual reassessment method (mCRM) with escalation with overdose control (EWOC) design (*n* = 280) (2) RO7121661 administered in treatment cycles once every 3 weeks. Dose escalation will be carried out according to a mCRM with an EWOC design (3) Cohort will comprise of participants with checkpoint inhibitor experienced, second line and beyond metastatic melanoma. The starting dose of RO7121661 for dose expansion will be derived from the maximum tolerated dose/recommended dose for expansion and the best dosing schedule determined during dose escalation.	1	Recruiting
*-*	NCT03489343	Advanced solid tumours or lymphomas	(1) Sym023 will be administered at up to 7 planned dose levels.	1	Recruiting
*Anti-NKG2A*	NCT02671435	Advanced solid tumours	(1) Escalation with 5 dose escalation cohorts. Durvalumab + monalizumab (*n* = 501) (2) Dose expansion with 4 dose expansion cohorts. Durvalumab + monalizumab (3) Dose exploration with 10 dose exploration cohorts. Durvalumab + monalizumab + standard of care systemic therapy ± biologic agent + monalizumab in combination with biologic agent in CRC	1/2	Recruiting
*Adoptive cell therapy*	NCT03007823	Small metastatic melanoma	(1) 8-10 billion high activity NK cells x3 intravenous (I.V) infusions (*n* = 10) (2) No intervention	1/2	Recruiting
*-*	NCT00846833	Malignant melanoma	(1) Cyclophosphamide then high dose IL-2 and NK cell infusion (*n* = 12)	1/2	Completed
*-*	NCT03420963	Malignant solid paediatric tumours	(1) Mesna dose prior to cyclophosphamide then 3 and 6 hours after each dose for a total of at least 80% of cyclophosphamide dose. 500 mg/m^2^ cyclophosphamide by vein on days -7 to -3. 100mg/m2 etoposide by vein on days -7 to -3. Participants receive IV NK cell infusion on day 0 (*n* = 32)	1	Recruiting
*-*	NCT00328861	Advanced melanoma or kidney cancer	(1) Cyclophosphamide 60 mg/kg/day I.V on days -8 and -7. Fludarabine 25 mg/m^2^ day I.V on days -6 through -2. IL-2 720,000 IU I.V every 8 hours for up to 5 days. 30 min infusion of NK cells 2 days after last dose of chemotherapy (*n* = 8)	2	Completed
*Cytokine*	NCT01727076	Advanced solid tumours	(1) Subcutaneous injection of recombinant IL-15 on days 1-5 of weeks 1 and 2. Treatment repeats every 28 days for up to 6 courses in the absence of disease progression or unacceptable toxicity (*n* = 20).	1	Completed
